# Dr. Chapman's Elements of Therapeutics and Materia Medica

**Published:** 1823-12-01

**Authors:** 


### *Elements of Therapeutics and Materia Medica.* By N. Chapman, M.D. In two volumes, 8vo. Vol. II.

[Concluded from No. 13, page 77.]

I. *Emmenagogues.*—The first section of the second volume of Dr. Chapman's work is on Emmenagogues. It is a general complaint, that the operation of medicine designed to act on the uterus is very precarious. Our author thinks that our disappointment in such cases does not so much result from want of power in these medicines to produce specific impressions on the uterus, as from the incorrect views which we have hitherto entertained of the process of menstruation, and still more from our overlooking the very different states of the system with which the suppression of the catamenia may be connected. Our author thinks that the process of menstruation is "very generally" considered to be a "mere effusion of blood." Dr. Chapman therefore undertakes to prove that this opinion is erroneous. Here our author combats a phantom, for we do not know a single practitioner or writer who now entertains such an absurd doctrine. Every body, in the present day, looks on the discharge as a secretion—as much so as milk from the mamma. This was John Hunter's opinion, as may be seen in the following quotation in Johnson's Midwifery, and is the generally received opinion of the profession at present. "'The blood discharged in menstruation, is neither similar to blood taken from a vein of the same person, nor to that extravasated by an accident in any other part of the body: but is a species of blood changed, separated, or thrown off from the common mass by an action of the vessels of the uterus, in a process similar to secretion, by which action, the blood, having lost its vital principle, does not coagulate.'" P. 6.

Our author now proceeds to the individual articles which have been found to produce a local action on the secretory function of the uterus.

1. *Polygala Senega.* To Dr. Hartshorne of Philadelphia, our author attributes the credit of first discovering the "admirable properties of this article as an emmenagogue." Dr. Chapman has made extensive trial of the medicine both in his public and private practice, "and with sufficient success to warrant him in recommending it as one of the most active, certain, and valuable of the emmenagogues." It may be used either in powder or decoction; the latter he prefers.

"In making the decoction, I put a pint of boiling water, to an ounce of the root, bruised, in a close vessel, and let it slowly simmer over the fire till the quantity is reduced one-third. Where the medicine excites nausea, which it is sometimes apt to do, even in small doses, I have it prepared with the addition of an aromatic, such as orange peel, or cinnamon." 8.

He prescribes about four ounces, more or less, daily, increasing the dose, about the expected period of menstruation, as far as the stomach will bear. In the intervals of the catamenial periods, he intermits the medicine for a week or two, lest it become nauseous or disgusting to the patient.

2. *Savine.* This is a warm, powerful, and diffusible stimulant, and seems to take a specific determination to the uterine system. Cullen spoke slightingly of it, and it has fallen into disuse—unmeritedly, our author thinks. The dose of the dried leaves is fifteen or twenty grains. Nearly the same observations apply to the rubia tinctorum, of which the late Professor Barton speaks in favourable terms. It it well known also, that the pulegium, or pennyroyal, is a very popular remedy for the purpose in question—and probably not without some foundation in fact.

3. *Secale Cornutum.* The Ergot is an elongated excrescence which fills the place of the seed within the glume or husk of rye. It is of an irregular curved form, something like the spur of a cock, of a dark colour, unpleasant taste, and brittle texture. Experiments have been made with it on animals, and it has produced a gangrenous state of their extremities. It is, therefore, probable that the idea long ago entertained that it produced a species of dry gangrene among people who fed on damaged rye, was not without some foundation in truth.

"As an article of the Materia Medica, the credit of establishing it, must be conceded to this country.* It is now eight or ten years since Dr. Stearns, of the state of New-York, gave to the world an account of its wonderful powers in promoting lingering labours. By a large number of practitioners it has since been tried with the same view, and though there may be some difference of opinion in relation to the degree of its utility, we have the most ample attestations in its favour. My own experience with it, which is by no means narrow, enables me to speak positively of its powers in this respect; and the same impression is entertained of it by several of

* "As a remedy in the promotion of labour, it was, no doubt, earlier employed in France. But the practice seems not to have been regulated with any propriety, and ceased after a short time."

my medical friends, engaged in the practice of midwifery in this city. I have been supplied with some very strong facts of a similar purport by several of my correspondents, and still more might be collected from the medical journals of our country. Though its powers, in this respect, seem to be sufficiently admitted, some discrimination is required in the selection of appropriate cases, as well as caution, in the use of the article.

"The mode in which I have been accustomed to prescribe it, is to dissolve a drachm of the powder in a small portion of water, and to give one-third of it every half hour, till the effect is induced. Not more than ten or fifteen minutes elapse before the uterus is excited into strong and violent pains, which, in all cases that have come under my notice, continued with little or no intermission or abatement, for a very short time, and then entirely ceased." 17.

It will be prudent, before using so violent a remedy, to ascertain that the os tincæ is pretty well dilated; otherwise we shall expose the woman to much pain, or perhaps to a rupture of the uterus. Our author, though he has used and seen it used, in more than a hundred cases, has never had reason to suppose that it acted deleteriously on the fœtus. It has slender powers as an emmenagogue. But, in some painful forms of dysmenorrhœa, he has seen it afford much relief by bringing away the deciduous lining of the uterus.

4. *Melampodium*—Cullen, with his usual scepticism, nearly proscribed this medicine in amenorrhœa: but it has many just pretensions, in Dr. Chapman's opinion, and especially when it purges. Thirty or forty drops of the tinctura melampodii constitute a dose every three or four hours, so long as the stomach will bear it without nausea or vomiting.

It is hardly necessary to remark, that the state of the system is to be taken into consideration. Some will require steel and other tonics—some will require the cold bath—many the warm hip-bath and local or general evacuations of blood.

"Nor is it by any means uncommon to meet with suppression of the menses, where the pulse is active, the habit plethoric, with a flushed face, and even eyes inflamed, accompanied by pain in the back and head, and with acute and uncomfortable sensations of fulness in different parts of the body. No one could hesitate a moment under such circumstances, to resort to venesection as the most prompt and effectual remedy. While it unlocks, as it were, the secretory actions, it obviates the danger which is too apt to ensue from vicarious hæmorrhages in the brain, the lungs, or some other vital organ. I have more than once seen, after a copious bleeding, the menses very speedily flow, and the obstruction permanently removed. Nor should we neglect, as auxiliary means, to invite the circulating fluids to the lumbar region, by the interposition of active purgatives. As operating on the large intestines which lie in the vicinity of the uterus, the various preparations of aloes have been much celebrated. There can be no doubt of their superior efficacy. Combined either with calomel or myrrh, aloes almost always displays active emmenagogue powers.* With the same view of causing a determination to the parts, I have sometimes employed, with marked advantage, blisters, and especially when applied to the inner part of the thighs." 23.

In respect to dysmenorrhœa, our author coincides with Dr. Dewees in supposing that it frequently depends on an imperfect secretory action of the uterus, by which an extraneous membrane is sometimes produced. Of the volatile tincture of guaiacum in these cases he has not yet had much experience.

"The remedy, however, in which I repose the greatest confidence in painful menstruation, is a combination of opium and camphor. I resort to it habitually, and scarcely ever without success. It is to be exhibited in the proportion of two grains of the first to ten of the second article; and if one dose should not answer, a second may be employed. Cases will occasionally occur where the sufferings are so severe as to demand the most immediate relief, and, under such circumstances, an anodyne injection sometimes operates very efficaciously." 24.

To do away with that state of the uterus on which this particular form of amenorrhœa depends, he recommends the suggestion of Dr. Dewees, already alluded to. "After all, we are not to lose sight of mercury in this disease. By a moderate salivation, continued for a week or two, we shall sometimes succeed in curing amenorrhœa, in its different forms, when every other mode of treatment has failed." 25. But, as Dr. Chapman observes, in the treatment of amenorrhœa, we must be prepared for disappointment. The secretion of the menses is probably much influenced by the state of the ovaries, and these are often disordered. By carefully examining the state of the system, however, and adapting our remedies to its condition, we shall generally succeed.

---

* "Not the least valuable, however, of these combinations is one known by the name of Hooper's pills. These are made according to the annexed prescription:

"℞. Sal. mart. ℥ss. Pulv. jalap ʒi. Hiera picra, ℥ss. Myrrh. ʒss. Syrup. com. q. s. ft. mass. div. in pil.

"Of these pills, which are prepared of a convenient size, two or three are taken at bed time, for several nights successively. They purge actively, and I presume it is by virtue of this property, that they operate so beneficially as an emmenagogue. In the original formula, I believe castor was added. The apothecaries of this city, however, have been induced to omit it, as rendering the pills offensive, without at all increasing their efficacy."

II. *Expectorants.*—These have been defined "those medicines which facilitate or promote the excretion of mucus or other fluids from the pulmonary system." There is much difficulty in accounting for the operation of this class of medicines, and there are many physicians of the present day who deny any such power as these are supposed to possess. Cullen conjectured that they increase the effusion from the arteries of the lungs, which being a thin fluid, dilutes the mucus of the pulmonary follicles, and by rendering it less viscid and tenacious, makes its expulsion more easy. Expectoration, in fact, may be promoted by very different means. Where an inflammatory condition of the mucous membrane obtains, bleeding will promote expectoration—and so will antimony or ipecacuanha, by lessening the force of the circulation, and producing nausea. When the lungs are oppressed with viscid secretion, and the constitution enfeebled, stimulants, as squill and gum ammoniac, will relieve the chest.

"But in all cases, and in whatever manner these medicines are productive of advantage, it is by an impression first made on the stomach, and subsequently extended to the lungs, through the medium of that sympathy which so intimately and conspicuously connects these parts. Nothing is more absurd, or less capable of being reconciled with the present state of our knowledge, than the hypothesis which is to be found, even in the most recent of the writers on the materia medica, that expectorants operate by reaching the lungs through the circulation!" 31.

Our author lays down only two rules for our guidance in the exhibition of expectorants.

"1. As in the use of diaphoretics, let the patient be kept warm. There is a very close consent between the skin and pulmonary organs, and we shall generally perceive that moderate warmth, and even moisture, on the surface, greatly facilitates expectoration.

"2. Carefully avoid purging. As remarked on a former occasion, none of the complaints of the lungs will bear this evacuation to any extent. Besides which, the action of the secretory vessels of the lungs and intestines, would seem to be alternate and opposed. Expectoration, at least, is very apt to be suppressed or diminished by diarrhœa or by the use of purges." 31.

Our author next descants on the individual expectorants, omitting the emetic substances, which are amply treated of under other heads.

The lichen islandicus, glycyrrhiza, mimosa nilotica, ammoniacum, scilla, allium, assafœtida, and polygala senega, pass in review; but, excepting of the latter, we need not here speak.

"This," says Dr. Chapman, "is one of the best of the expectorants. To pneumonia, under almost all circumstances, it is, at least in popular practice, applied, and the confidence reposed in it, in some parts of our country, has long been, and still continues, exceedingly high. Considering, however, its stimulating nature, it ought not to be thus indiscriminately employed. Excepting typhoid pneumonia, it is inadmissible in the early stages of the acute complaints of the chest. But after inflammatory action has been reduced by previous depletion, it comes in exceedingly well as an expectorant, and, perhaps, also, as having a tendency to relieve congestion of the lungs, by promoting determinations to the surface. It is in this way that it operates so beneficially in the pneumonia of infirm people, which is always attended with debility of the pulmonary organs, and, consequently, with large and oppressive lodgements of phlegm or mucus." 43.

Our author considers, and with reason, that the ammoniæ carbonas is among the most important of expectorants, especially in the advanced stages of acute pneumonia. But more will be said of this medicine hereafter. Passing over colchicum we come to the balsams, and we extract the following note respecting the copaiba.

"I have lately learnt from Mr. Brown, a most intelligent apothecary of this city, a much more convenient mode of exhibiting this medicine, than any hitherto proposed. The copaiba is poured on half a wine-glass full of water, and immediately afterwards are slowly added, in the same way, a few drops of the common bitter tincture. By this, the copaiba is collected in a small globule, readily swallowed, and its taste, so nauseous to most people, is entirely lost in the bitterness of the vehicle. Given in this mode, it always rests well on the stomach." 50.

As an inhalation in dyspnœa and other chronic affections of the lungs, our author mentions a combination of a teaspoonful of Hoffman's liquor and the same quantity of laudanum, put into an inhaler with some boiling water and the steam breathed. He thinks the effects of the smoke of stramonium have been greatly exaggerated.

The section on anthelmintics in Dr. Chapman's work, offers nothing for quotation into our pages. A section follows on blisters, which contains much useful practical information, but which we shall also pass over.

The application of blisters to particular diseases next follows in the work under review; but the observations though judicious, contain nothing novel, if we except the following passage.

"Of the numerous improvements in surgery which have been suggested by Dr. Physick, there is scarcely any one of more unequivocal utility, than the management, by blisters, of inflamed veins from the operation of bleeding. In most cases, a single one will prove adequate to the cure, though occasionally, where the arm is very stiff and tumid, a succession of them is demanded. The mode of application is, to confine a strip of the plaister along the whole course of the inflamed vein, making an opening for the pus or sanies to escape." 113.

III. *Diffusible Stimuli.* The 7th section is on diffusible stimulants. Our author observes, in limine, that, while he maintains the incitant or stimulating effect of most substances applied to the human body, he still is not among those who insist that they are all endowed with the same properties, differing only in degree of force, permanency, and diffusibility. "Consistently with his own conviction, there are no two articles which produce precisely the same effects." In this we perfectly agree with our ingenious author. There is a distinction in regard to general stimulants which is too important to be overlooked. We have one set distinguished by great diffusibility, and which nearly as soon as exhibited occasion universal excitement over the body—and there is another class, by which tone is imparted, though very slowly, and only by a long continued administration. The diffusible are very transient in their effects; the others produce permanent or enduring impressions.

Respecting narcotics, it is not necessary to enter into the dispute about their directly or indirectly stimulant properties. Most practitioners are acquainted with the circumstances which indicate and contra-indicate their employment, and that is the main point.

"Exhibited in a moderate dose, the purer narcotics excite activity both of body and mind. The force, frequency, and fulness of the pulse are increased, muscular action is rendered more vigorous, the temperature of the surface becomes higher, some of the secretions are promoted, and hilarity and animation of spirits are induced and kept up for a season. But the effects enumerated, as caused by a limited use of these articles, having continued for a short period, are succeeded by very unequivocal signs of diminished action, and subdued sensibility. The pulse becomes slower, and more full than natural—the secretions, except by the skin, are abated—there is less susceptibility to impressions—pain is alleviated, and inordinate motions repressed—muscular power is abridged, and the mind, partaking of this general langour, is dull and inactive. This state either terminates in sleep, or produces a tendency to it.

"By a large dose, debility, without any previous excitement, is occasioned, or if there be excitement, it is so evanescent, as not to be perceived—and when the effect goes off, there are tremors, sickness, head-ache, and oppression.

"Taken in excess, the system at once sinks under the impression, and the consequences are either wild delirium, or heavy stupor, deep, difficult, and stertorous respiration, convulsions, apoplexy, or paralysis, and ultimately death." 145.

It is thus manifest that most of the narcotic class are capable of producing stimulant or sedative effects, according to the mode in which they are administered. To command their stimulant power, they ought to be prescribed in small doses, frequently repeated and gradually increased—by which the excitement they enkindle is sustained. But when the design is to mitigate pain, to procure sleep, to relieve irritation, or deaden sensibility, they should be exhibited in a full dose, and at more distant intervals. It is not easy, as Dr. Chapman observes, to fix the period, in the progress of a disease, or to ascertain the circumstances in which it becomes proper to prescribe stimulants. It is far more easy to come to a just decision in respect to the use of depleting measures.

"Meaning, in the administration of stimulants, to overcome an existing action, by exciting a new and a stronger one, it is obvious that they can only be resorted to with any hope of advantage in the feeble shapes of disease, or in more violent forms reduced by previous evacuations. In determining the exact point at which to commence the use of stimulants, we may also be aided by watching their operation. Being ill-timed, they commonly produce pain in the head, or delirious wanderings, or morbid vigilance, or stricture of the breast, or restlessness and anxiety with a hot dry skin, parched tongue, and a quick, small, and corded pulse." 147.

In the arrangement of particular *stimulants*, our author has been a little embarrassed; for practical convenience he thinks they may be all brought under the three heads of *incitants*, *narcotics*, and *antispasmodics*. Under the first head are placed carbonate of ammonia, camphor, ol. terebinthinæ, phosphorus, piper indicum, caryophylata, cubebs, ginger, alcohol. Under the head of narcotics, we have opium, lactucarium, tela areneii, hyoscyamus, conium, belladonna, solanum nigrum, solanum dulcamara, stramonium, prunus lauro cerasus, digitalis, nux vomica. Under the head of antispasmodics he places; musk, castor, assafœtida, valerian, allium sativum, ol. succini, ol. cajuputii, ether. On each of these Dr. Chapman makes many judicious observations, at some of which we shall glance.

*Camphor*. Speaking of that very curious and often fatal disease, delirium tremens, Dr. Chapman makes the following remark.

"Without entering into any disquisition relative to the nature of that species of mania excited by intoxication, I shall remark, that the approved plan of managing it, consists in the occasional use of emetics, and in the steady exhibition of the most powerful stimuli. Even though it may seem to be forbidden by contra-indications, we ought not to depart from this course. The symptoms of vigorous and inflammatory action incident to the case are always fallacious, and the system, for the most part, very speedily sinks into a dangerous degree of debility by the slightest depletion.

"Of all the remedies which I have ever tried, combinations of camphor and opium are the most successful in this disease: and it is, indeed, not easy to conceive of any mode of practice more efficacious. My rule is to give these medicines in large doses, till the patient becomes composed, and then to sustain his strength by a generous diet, and cordial drinks. The preceding remarks have reference more particularly to cases occurring with habitual drunkards, and where the constitution is shattered, and broken down." 171.

We lately examined the body of a gentleman in the prime of life, and of very strong constitution, who fell a victim to this disease. Though he had all the *fallacious* symptoms of phrenitis—red ferretty eyes, throbbing of the carotids and temporals, dry tongue, and raging delirium, there was not a single trace of vascular fulness of the brain, and we had the assistance of Sir Astley Cooper, Mr. Freeman, and one or two other medical gentlemen, in examining it. We found, however, some earthy depositions in the falciform process, and very many spiculæ of bone rising from the basis cranii. But after all, these could only have been predisposing causes. The ingurgitation of inebriating materials was the exciting cause of the disease.

*Oil of Turpentine.* More than 20 years ago Dr. Physic of Philadelphia, was in the habit of employing this medicine in the grave fevers of that climate. Dr. Chapman in the year 1820, being convinced from dissections as well as symptoms, that the endemic yellow fever of the United States, "consists in a peculiar inflammation of the stomach, caused by the action of effluvia upon it," instituted a new practice, in conjunction with Dr. Hewson, at the Public Hospital of Philadelphia.

"After moderate evacuations of the bowels, we exhibited the turpentine, in doses adapted to the emergency. Commonly a drachm was given every hour or two, sometimes alone, and at other times with carbonate of ammonia, or some essential oil, which rendered it less disagreeable to the taste, and made the stomach more retentive of it.

"Of sixteen cases managed in this way, twelve recovered. Compared with what was done in the city by other modes of treatment, this success is exceedingly encouraging. It should, too, be recollected, that the patients were brought into the hospital, very often, in an advanced stage of the disease, and when much reduced by venesection and other evacuations. Unless it be applied at the commencement, or very early in an attack, the turpentine, in common with all remedies, will be, for the most part, unavailing." 175.

As the idea of gastritis cured by so stimulating a remedy as oil of turpentine, might appear to involve a degree of incongruity, our author has entered into some explanation on the subject. He properly enough observes that nothing is more delusive than the doctrine of identity of diseased action, or of remedial agency.

"We cure some inflammations by reduction with the directly depleting measures, while others are overcome by counteraction, at once subverting the morbid movements which are going on at the time, in a part, or the whole of the system.

"Do we not see this in the efficacy of certain stimulating collyria in ophthalmia,—in the copaiba and cubebs in gonorrhœa—in the eau medicinale in gout—in mercury in syphilis,—in the capsicum in cynanche tonsillaris—in mercurial ointment in erysipelas,—not to adduce other instances, which might be done by appealing to the records of the Brunonian practice, which was often successfully conducted on this principle?

"Of the *counter-agency* of turpentine in scalds and burns we are aware. The stomach, in yellow fever, is in a state of inflammation, probably of a somewhat similar nature, which is overcome in the same way. This conjecture derives support from the consideration, that, in many instances, the turpentine is soothing in its effects, removing the sense of heat and irritation in the stomach, subduing the force of vascular action and general excitement, and inducing, at once, a condition altogether of more comfort and security. Nor is it to be overlooked, that, without any previous intercommunication of opinion, the turpentine was prescribed, with great advantage, nearly about the same time, in the plague at Malta,* and is strenuously recommended as the best corrective of the inflammation of the stomach from the acrid poisons,† two cases, in many points, approaching so closely to yellow fever. These coincidences are very striking, and in every view deserve our most serious attention." 176.

Still our author does not propose this remedy confidently in yellow fever. He believes that the more violent forms are irremediable under any treatment.

He has been in the habit of employing oil of turpentine in peritoneal inflammation for many years past with decided success; and he thinks it may be equally useful in enteritis.

*Alcohol.* Moderately used, this is one of the least equivo-

* "Calverts on the Plague." "† Orfila on Poisons."

cal of the purely cordial and exhilarant stimulants; but urged to any extent it loses this power, and produces dulness and stupefaction—hence it is first a stimulant and subsequently a narcotic. Its effects indeed on the human frame are too familiar to need description. As a medicine, it is rarely necessary, for there are but few cases in which it is preferable to wine, *per se*. It is a sacred, but almost a needless duty in the physician to unite with the moralist, the divine, and the economist, in reprobating the consumption of this baneful article. Chained, by a species of infatuation to the use, or rather the abuse of these intoxicating beverages, as fast as Prometheus to his rock, mankind have hitherto seemed equally heedless to the admonitions of the wise, and to the suggestions of their own understandings in this respect; and so great is the moral as well as the physical mischief, that the emptying of Pandora's box was but the type of what has since happened in the diffusion of rum, brandy, gin, and whiskey, among the human species. This evil exists to a still greater extent in the new than in the old world. Poverty among the lower classes restrains the indulgence in this country, where the materials are such an enormous price —and public opinion has greatly checked inebriety among the upper and middling ranks of life. In America, on the other hand, more money is earned by the working classes of society, and spirituous potations are not one third the price they bear in England. With such temptations in their way, intoxication must ever be a darkening feature in the complexion of the common people of the new world.

"Compared with ardent spirits, the action of wine is infinitely less injurious in a state of health; and, as a remedy in disease, it evinces the same superiority. The effect it produces is slower and more permanent, combining also qualities, which, while they blunt the ardency of the stimulus, afford no inconsiderable portion of nutriment, by which the system is sustained and invigorated." 196.

Since the downfal of Brunonian principles, medical men rarely venture on the use of spirituous or vinous stimulants in febrile diseases, unless the type be of a very bad kind—and even then, not at the beginning of the disease, but to assist the powers of nature towards the crisis or close of the malady.

"As the excitability of the system in these cases is nearly expended, a very copious exhibition of wine is generally demanded, in order to attain its beneficial effects. It is sometimes drunk in quantities so large as to be incredible, were we not acquainted with the fact which I have mentioned, of the great insusceptibility to its impression. Nevertheless, even here, we are cautiously to regulate its administration by the effects it manifests, since, urged too far, it might induce indirect debility, and thus cause irreparable mischief." 197.

Rum may always be considered as beneficial if it render the pulse fuller, slower, and stronger—when it lessens or removes delirium, calms irritation, and induces sleep, and *vice versâ.*

*Opium.* Medical sentiment still continues rather divided on the modus operandi of opium, "though the preponderance is decidedly in favour of its stimulating effects." From a number of experiments made on opium by our author and others, the following results have been deduced.

"It appears that "opium applied to the eye, internal membrane of the nose, urethra, or other similar sensible surface, or to any other part of the body, deprived of its cuticle, is first productive of pain, a sense of heat, and of inflammation, and after the cessation of which symptoms, the natural, or morbid sensibility of the part is diminished.

"Exhibited internally, in an adequate dose, opium produces the following changes in the *vital functions.*

"The pulsations of the heart and arteries are first rendered quicker, fuller, and stronger, and afterwards slower than at the time of taking it. With the increase of frequency in the pulse, the heat of the body is generally somewhat augmented. The respiration is little affected, except a large dose has been taken, towards the conclusion of the operation of which, it becomes slow, stertorous, and laborious.

"The *natural functions* are thus disturbed. The appetite and digestion, from unusually large, or frequently repeated doses, are generally impaired, and vomiting often induced : the discharges from the intestines are diminished or suppressed, secretion and excretion are impeded in every part of the system, except the skin, the discharge from which is evidently augmented, sometimes preceded or attended with a sense of pricking or itching of the skin, terminating now and then, in a species of miliary eruption.

"The *animal functions* are affected as follows :

"The hilarity of the mind is by degrees augmented, and continues to increase, if the dose be considerable, until the delirium of intoxication is produced, which, as when resulting from spirituous liquors, is attended in different constitutions with different symptoms. It is, however, more generally productive of a pleasant and joyous state of the mind than the contrary, and, in many, it occasions an increased disposition to venery. After these effects have continued for some time, they are succeeded by others of a very opposite nature : the mind becomes gradually dull and languid, the body averse to motion, little affected by customary impressions, and inclined to sleep. If the dose has been considerable, all these symptoms continue to increase, and tremors, convulsions, vertigo, stupor, insensibility, and deprivation of muscular action, appear variously complicated, and in various degrees, proportioned to the excess of the dose, and peculiarity of constitution in the sufferer." 204.

On the application of opium in particular cases of disease we need not dwell. Dr. Chapman's directions and observations are very generally judicious.

*Tela Aranei.*—It is a very old popular belief that the spider's web cures agues. But among medical men, till within the last twenty years, little credit was given to this popular superstition as it was thought. In Dr. Robert Jackson's last visit to Philadelphia he mentioned the efficacy of the remedy as indisputably ascertained, and averred (what he afterwards published) that, as an anodyne to allay pain or calm irritation, it proved vastly superior even to opiates. Dr. Broughton, of South Carolina, formerly one of Dr. Chapman's pupils, experimented on the substance in question, and confirmed, in all material points, the statements of Dr. Jackson. As the writings of this able and zealous physician are not sufficiently diffused among our professional brethren, we shall here introduce a quotation on the subject in question from one of his works.

" 'I think I may venture to say, that it prevents the recurrence of febrile paroxysms more abruptly, and more effectually than bark or arsenic, or any other remedy employed for that purpose with which I am acquainted: that, like all other remedies of the kind, it is only effectual as applied under a certain condition of habit; but that the condition of susceptibility for cob-web is, at the same time, of more latitude than for any other of the known remedies. The cob-web was rarely given before the subject was prepared by bleeding, emetics, or purgatives; and, given to a subject so prepared, it seldom failed to effect a cure comparatively permanent; relapse, or conversion into another form of disease, being, upon the whole, a rare occurrence where the disease had been suspended by this remedy. If the cob-web was given in the time of perfect intermission, the return of the paroxysm was prevented; if given under the first symptoms of a commencing paroxysm, the symptoms were suppressed, and the course of the paroxysm was so much interrupted, that the disease for the most part lost its characteristic symptoms. If it was not given until the paroxysm was advanced in progress, the symptoms of irritation, viz. tremors, startings, spasms, and delirium—if such existed as forms of febrile action, were usually reduced in violence, sometimes entirely removed. In this case, sleep—calm and refreshing, usually followed the sudden and perfect removal of pain and irritation. Vomiting, spasms, and twisting in the bowels, appearing as modes of febrile irritation, were also usually allayed by it: there was no effect from it where the vomiting or pain was connected with real inflammation or progress to disorganization. In cases of febrile depression, deficient animation, and indifference to surrounding objects, the exhibition of eight or ten grains of cob-web was often followed by exhilaration;—the eye sparkled; the countenance assumed a temporary animation; and, though the course of the disease might not be changed, or the dangers averted, more respite was obtained from a pill of cob-web than what arises from, or belongs to the action of wine, opium, or any thing else within my knowledge.

" 'Further, the power of cob-web has been tried, and its good effects have been proved, in other forms of irritation besides those that are strictly febrile. In spasmodic affections of various kinds, in asthma, in periodic head-aches, in general restlessness and muscular irritabilities, its good effects are often signal. The cob-web gives sleep, but not by narcotic power:—tranquillity and sleep here appear to be the simple consequence of release from pain and irritation. Cob-web has also been applied locally,—under my own eye, to ulcerated and irritable surfaces with singular good effect. At first, the pain which it occasioned was sharp,—but it was momentary; and the surfaces, which had been painful, irritable, and untractable to other applications for weeks or months, were healed up in the course of two or three days at farthest:—the experiment was made on superficial sores only.

" 'I have not made a chemical analysis of the substance in question; for my chemical knowledge is not of the kind which would enable me to conduct the operation correctly, The cob-web may, perhaps, be thought to belong to the class of poisons; but, it is somewhat singular, that I have not been able to discover much difference of effect from a dose of ten grains and from a dose of twenty. The changes induced on the existing state of the system, as the effect of its operation, characterize it as powerfully stimulant. 1. Where the pulses of the arteries are quick, frequent, irregular, and irritated; they become calm, regular, and slow—almost instantaneously after the cob-web has passed into the stomach: the effect is moreover accompanied, for the most part, with perspiration and perfect relaxation of the surface. 2. Where the pulses are slow, regular, and nearly natural; they usually become frequent, small, irregular,—sometimes intermitting. 3. Where languor and depression characterize the disease; sensations of warmth and comfort are diffused about the stomach, and increased animation is conspicuous in the appearance of the eye and countenance. 4. The cob-web, applied to a bleeding surface, occasions a very sharp and transient pain:—the bleeding instantly ceases.—The cob-web here recommended is the produce of the black spider which inhabits cellars, barns, and stables: that which is found upon hedges in autumn does not possess the same power, if it be actually of the same nature.' " 241.

The spider's web has been, for some time past, pretty liberally prescribed by Drs. Physic, Chapman, and Dewees in Philadelphia; and though they attach different *degrees* of value to the article, "they are all satisfied that the representation of its virtues (to which we have referred) is very little, if at all exaggerated." In doses of five grains, repeated every fourth or fifth hour, Dr. Chapman has cured some obstinate intermittents, suspended the paroxysms of hectic, overcome morbid vigilance from excessive nervous mobility, and quieted irritation of the system from various causes. That used by our author was collected in cellars, and was probably the product of the common black spider. He has also satisfied himself that the web found in light exposed situations, the product of the grey spider, is inert—and so is the web of the other, when not recent, which may be known by its glutinous feel.

Passing over a number of narcotics, and among the rest the prussic acid, which our author observes has come forward "with bloated promise and lank performance," we come to the class of antispasmodics, but Dr. Chapman's remarks on these need not detain us.

*Hydrargyrus.* The eleventh section is on the various preparations of mercury. We shall here quote a passage from this section, as it bears on a recent investigation carried on for a considerable time, with some clashing of sentiment, in this metropolis.

"As a part of the treatment of dysentery, the mercurial medicines have been deemed very important. By Cleghorn, who acquired so much reputation by his work on the diseases of Minorca, six or eight grains of calomel, with one of opium, were exhibited with a view to purge, and, he declares, that it answered more effectually than any other measure. I have already mentioned* the advantages derived from large doses of opium and calomel in those unrelenting obstructions of the bowels dependent on spasm: and it is quite conceivable, that, on the same principle, such a combination might be useful in certain cases of dysentery.

"Cleghorn, as I have stated, originally prescribed calomel and opium as a purgative; but he was ultimately convinced of the superior efficacy of salivation in these cases. This taking place, he even asserts that a cure invariably followed. The practice was nearly as successful in the hands of Lind, in the West, and was found highly so by Balfour, and by Yeates and Maclean, in the East Indies.

"Having in another place detailed my mode of managing this disease, I have now little to say. Combinations of calomel, ipecacuanha, and opium, with a view of quieting irritation and determining to the surface, have, at all times, been a favourite prescription of mine. To this I shall only further remark, that in protracted or chronic dysentery, I have frequently salivated, and found it sometimes the only means by which I could effect a cure. Yet, in the acute forms of the disease, I should presume that it can hardly be expedient to resort to this unpleasant alternative; though, it is probable, that whenever salivation is excited, it will have a beneficial tendency.

---

* "Cathartics, opium, &c."

"This opinion is applicable chiefly to the disease as it appears in this city. No doubt, in warmer climates, as in the East and West Indies, and in the southern sections of our own country, where the hepatic system is deeply implicated in this and in most other affections, the mercurial treatment is much more frequently demanded. Dysentery among us is an inflammatory affection, calling, in the first instance, for the prompt and free use of all the direct depletory measures. To this general character of the disease there is one exception. In our poor and depraved classes of society, it now and then assumes the typhoid state, and such is generally the case when it breaks out in crowded and ill-ventilated apartments. Depletion, under such circumstances, cannot be urged to any extent, and I have early put the system under the mercurial impression, with great effect.

"Mercury, as a dernier resource in diarrhœa, should not be overlooked. Cases of a very lingering and obstinate nature I have repeatedly seen yield to this remedy. It is, indeed, almost indispensable where indications exist of derangement in the chylopoietic viscera: and even if this be not the case, it proves of service by instituting a new set of actions in the intestines." 329.

In dyspepsia our author follows the Abernethian practice; and he alludes to a particular form of this complaint which he believes to depend "on a slow species of inflammation of the stomach," not confined to intemperate livers, but affecting also people of the most regular habits.

"Cases of this sort may be distinguished, by the strong sensations of heat and pain which are felt in the stomach,—by tenderness on pressure of the epigastrium, and by a pulse hard, corded, and quick. There is also, after a while, an irregular, diminutive, hectic fever, with suffusions of the cheeks, yellowness of complexion, a hard dry cough, pains in the side, and some wasting of flesh and strength. The condition, in short, has so many of the aspects of the incipient stage of tubercular consumption, as to be very readily confounded with it. I have indeed known it, sometimes, to induce pulmonary disease of the most serious character. An irritation of the stomach, long continued, will sympathetically extend itself to the lungs, whether it be from sordes, worms, indigestible substances swallowed, or any other cause. Cases illustrative of this principle have been repeatedly recorded, and must have come under the notice of almost every practitioner." 330.

Venesection is recommended in this form of dyspepsia, with very minute doses of ipecacuanha. "This failing, mercury may be added, or used alone to the point of moderate salivation which, with a blister to the epigastrium, and a duly regulated diet, I have found generally succeed." We are induced to give the following extract, as it bears on another legal investigation of medical practice, in which we conceive one of our brethren (Dr. Veitch) was very illiberally, not to say unjustly, treated.

" To treat *mania* by the mercurial medicines, if not an American practice, is certainly much more prevalent among us, than in Europe. It is one, at least, to which the medical men of this city are exceedingly devoted. Yet, I do not think that we have determined, with sufficient precision, the cases to which it is best suited, and hence the results of our experience are very different, and even contradictory. It is resorted to in each form of mental derangement, as well in furious mania as in melancholia, and, perhaps, with nearly equal success. All that is particularly attended to in the employment of the remedy is, to see that the system is properly reduced, or, in other words, brought down by bleeding, purging, and other evacuations, to the point of salivation. This being premised, mercury unquestionably will often effect cures under circumstances apparently the most discouraging. It is, in short, a principle with most of our practitioners to appeal to salivation as a resource in all cases of insanity, which have pertinaciously resisted other modes of treatment, and, though this may be empirical practice, it is fully justified by the frequency of its success.

" That the utility of mercury in the mental affections may in part be ascribed to its general powers over disease, seems highly probable. But I cannot help entertaining the conviction, that it does infinitely more good by specifically acting on the chylopoietic viscera, correcting the derangement in this system of parts, which would seem to be the cause, in very many instances of the morbid states of the mind. Extraordinary as this *location* of insanity may appear to such as have not contemplated the subject, it neither wants the support of authority, nor the evidence of analogy, or of positive facts. It is now pretty generally conceded, that the whole class of neuroses is very frequently of gastric origin. Considering the close affinity which exists in the physiology as well as pathology of the nerves and brain, it is surely no great stretch of generalization, to embrace within the same view the two sets of cases.

" Can it be denied that the cerebral and mental disorders are frequently induced or imitated by impressions on the stomach and its dependencies from the narcotic poisons, worms, and other causes? Every practitioner of much experience has probably seen this repeatedly illustrated in apoplexy, palsy, hydrocephalus, cephalalgia, in the depravation of vision, and in mania, melancholia, and hypochondriasis, all which is abundantly confirmed by dissection.

" It had long been a matter of surprise with pathologists, that, in the diseases of the mind, the morbid appearances of the brain should be so few and slight; sometimes, indeed, none whatever existing, even though the case were furious mania. Being conducted under the predominant notion that the brain must necessarily be the seat of these affections, examinations were rarely extended beyond this organ; and consequently, the real source of mischief continued unrevealed. But of late, the cultivators of morbid anatomy in Europe have thrown a very clear and distinct light on this subject, demonstrating incontestibly, that we are to seek in the chylopoietic viscera for the causes of many of the nervous and mental complaints. The facts thus developed, have laid the foundation of a correct pathology with respect to this interesting set of diseases, and will, no doubt, as has already in some measure been done, lead to a more exact and successful mode of treatment. But, while I maintain generally the connexion of these cases with certain disordered states of the abdominal viscera, I am not at all disposed to deny, that they may take place as idiopathic affections of the cerebral and nervous systems." 337.

In respect to syphilis, our author is satisfied, from wide experience, that mercury is to be preferred to every other remedy. And he properly observes, that a very moderate course is, in general, to be preferred to a rapid introduction of the remedy and a profuse salivation. But our author's practice has one peculiar feature which appears to us to involve great inconsistency, and hold out a dangerous precept. Chancre he treats entirely as a local disease, and by local remedies. He does not exhibit mercury for the chancre, but only when the disease appears in the throat, or other parts of the body besides the genital organs.

" Allowing, however, that this expedient (local application to the chancre) occasionally fails, and the system becomes affected, where is the increased evil or mischief? Let the patient, in other words, return to me after a few weeks, with an ulcerated throat, and other symptoms of confirmed lues, what happens? Entertaining, as I do, the conviction of the superiority of the mercurial treatment, I should instantly commence it, and with the assurance of effecting a cure as promptly, and with as little inconvenience, as if it had originally been employed. As, therefore, no more mercury is required to eradicate, than to prevent the complaint, there cannot be a comparison between the two modes of practice in these cases.

" But I am prepared to go further, and to avow my total want of confidence in the powers of mercury to cure chancre, or hinder the contamination of the system. My reason for thinking so is, that I have several times known a chancre take place, and extend its ravages as usual, though, at the time, the individual was fully under the mercurial impression. Such examples are by no means rare in the public institutions of great cities. Thus, we see patients, while in a deep salivation for dropsy, hepatitis, consumption, or some other complaint, contract chancre, followed by buboes, &c. Whether the disease, if not checked, would further extend itself, my experience does not allow me to say. The fact seems to me, that chancre, which is a mere local injury, is so seated on the confines of the system, that it cannot be approached, or at least very slowly, by general remedies, and must be managed with direct applications. No more, in my opinion, can we cure it by mercury exhibited internally, than suspend the career of the vaccine or variolous pustule, or disperse a paronychia, or heal any minute and ordinary sore" 356.

Thus, then, because Dr. Chapman has " several times known a chancre take place" while a man was under a mercurial course, he concludes, in the direct face of universal experience, that mercury has no power over primary sores! What practitioner has not, hundreds of times, seen a chancre remain perfectly stationary, or gradually enlarging, till the mercurial odour appeared on the breath, and from that instant put on a new and healthy appearance? And is this wide and universal experience to give way before a hypothetical fancy, or a few insulated anomalies?

Here we must close this article, as the two concluding sections of Dr. Chapman's work, on Tonics and Astringents, present nothing on which we could dwell with advantage. Our readers have been put in possession of ample materials and extracts from these volumes to enable them to judge for themselves respecting their merit, which is undoubtedly very considerable. There is as much originality as could possibly be expected, now-a-days, in Dr. Chapman's observations; and his didactic precepts are, with a few exceptions, remarkably judicious.

